# On Influence of Mechanical Properties of Gun Propellants on Their Ballistic Characteristics Determined in Closed Vessel Tests

**DOI:** 10.3390/ma13143243

**Published:** 2020-07-21

**Authors:** Radosław Trębiński, Jacek Janiszewski, Zbigniew Leciejewski, Zbigniew Surma, Kinga Kamińska

**Affiliations:** Faculty of Mechatronics and Aerospace, Institute of Armament Technology, Military University of Technology, 2 Gen. S. Kaliskiego Street, 00-908 Warsaw, Poland; jacek.janiszewski@wat.edu.pl (J.J.); zbigniew.leciejewski@wat.edu.pl (Z.L.); zbigniew.surma@wat.edu.pl (Z.S.); kinga.kaminska@wat.edu.pl (K.K.)

**Keywords:** gun propellants, mechanical material properties, high strain rate testing, shape function

## Abstract

The geometric burning law of gun propellants is widely used in computer codes used for the simulations of the internal ballistics of guns. However, the results of closed vessel tests prove that the burning process of some propellants deviates from the geometric law. Validation of the hypothesis that observed deviations can be attributed to the cracking of propellant grains was the aim of this work. In order to verify the hypothesis, three types of gun propellants were chosen with considerably differing mechanical strengths: a single-base propellant, a double-base propellant, and a composite propellant. The mechanical properties of the gun propellants were tested using a quasi-static compression method with strain rate values of the order of 0.001 s^−1^ and the Split Hopkinson Pressure Bar technique with the strain rate in the range of 1000–6000 s^−1^. The mechanical responses of the propellants were assessed on the basis of the true stress–strain curves obtained and from the point of view of the occurrence of cracks in the propellant grains specimens. Moreover, closed vessel tests were performed to determine experimental shape functions for the considered gun propellants. Juxtaposition of the stress‒strain curves with the experimental shape functions proved that the observed deviations from the geometrical burning law can be attributed mainly to the cracking of propellant grains. The results obtained showed that the rheological properties of propellants are important not only from the point of view of logistical issues but also for the properly controlled burning process of propellants during the shot.

## 1. Introduction

The pressure *p* (1) [1,2,3] generated by the combustion of a propellant charge in a gun system depends on the following factors:

- the parameters of the barrel: capacity of the chamber *Wo*, barrel cross-sectional area *s*, secondary works coefficient φ,
- the mass of the projectile *m_p_* and its velocity *v_p_*(*t*) attained at a given projectile path *l*(*t*); thermodynamic parameters of propellant gases: force *f*, covolume *η*, and ratio of specific heats *k* (θ = *k* − 1),
- the relative burned volume of propellant grain *z* (ratio of the burned volume to the initial volume), and
- the mass *ω* and the density *ρ* of the propellant, as well as the shapes and sizes of the propellant grains.

(1) $$p(t)=\frac{f\omega z(t)-\frac{1}{2}\theta \varphi m_{p}v_{p}^{2}(t)}{W_{0}+sl(t)-\omega \left[1-z(t)\right]/\rho -\eta \omega z(t)}.$$

The process of gas generation has a decisive influence on the pressure pulse. It can be described by the following Equation (2) [1,2,3], which relates the relative volume of burned propellant *z* and the relative burning surface *ϕ*, the burning rate *r* (expressed in m/s), the grain initial volume *V*_0_, and the initial surface area *S*_0_:

(2) $$\frac{dz}{dt}=\frac{S_{0}}{V_{0}}\phi \left(z\right)r\left(p\right).$$

The equation of gas generation is based, among others, on the following assumptions constituting the geometric law of burning [1,2,3]:

- all propellant grains are ignited at the same time,
- propellant grains burn in parallel layers,
- the burning rate is only a function of the pressure, and
- there is no cracking of grains.

Equation (2) is widely used in computer codes used for the simulation of internal ballistics of guns. However, the results of closed vessel tests prove that the shape function *ϕ*(*z*) may deviate considerably from the one based on the geometric law. The reasons for this are not clear. A hypothesis was formulated in [1] that the difference between the pressure values inside the perforation of propellants grains and outside of the perforation causes faster burning inside the perforation. It is equivalent to an apparent increase of the burning surface. So, deviations from the geometric burning law should be an inherent feature of burning of perforated grains. However, we show in this paper that it is not the truth. We present the results of our study on the validation of a hypothesis that the deviations from the geometric burning model can be a result of the cracking and the disruption of propellant grains. These can be caused by the impacts of grains against the walls of a case and the mutual collisions of grains. This can take place during the transport of ammunition and also during a shot. The cracking and disruption of propellant grains affect the pressure course *p*(*t*), and it can thus differ from the pressure course calculated theoretically. In extreme cases, the disruption of grains can strongly accelerate the pressure rise, which can cause damage to the launching system. So, it is very important to control the mechanical properties of gun propellants in the range of quasi-static loading conditions that is characteristic for the logistics of the ammunition, as well as at the dynamic loading range resulting from a very fast pressure rise during the shot.

In general, the mechanical properties of gun propellants are determined using a standard test technique that is widely used in engineering material studies. A very simple method of strength testing of propellant grains is described in [4]. A cylindrical isometric propellant grain is compressed by a manual press to the moment of the first crack occurring on the side surface. The strain corresponding to this moment is used as a characteristic parameter of the tested gun propellant. 

The influence of the mechanical deformation of ball single-base propellants on the characteristics of the burning process was investigated in [5]. The grains conditioned at various initial temperatures were compressed under 10 MPa for 1 min in a manual hydraulic press. Next, the compressed grains were tested (after thermal conditioning at ambient temperature) in a closed vessel. The results of these studies confirmed two phenomena at low temperatures: the first is the decrease in combustion rate as the initial temperature decreases, and the second is the grain cracking that occurs during ignition. 

Taking into account the dynamic character of loads during the shot, a need for high strain rate testing of propellants was recognized. In work [6], the results of compression tests with the strain rate of 10 s^−1^ were reported. A modified set of high-speed pneumo-hydraulic testing machines was used. In this case, the loading did not affect the propellant grains directly, but it was transferred to the propellant by a piston that enabled the measurement of the strain. A modified set, but using the same principle, was used in [7,8,9] for comparing the mechanical responses of a double-base JA-2 propellant, triple-base M30 propellant, and low vulnerability ammunition (LOVA)-type XM39 propellant. The range of strain rate values was broadened to 250 s^−1^. 

A drop weight testing machine was used in [10]. Damage to the propellant grains was analyzed. The stress‒strain curves were determined for a wide range of temperatures: from −60 °C to +50 °C. In works [11,12], the same technique was used for investigating the influence of the content of hexogen on the mechanical properties of composite propellants. An interesting testing method was used in [13]. Grains of JA-2 and M30 propellants were launched by the use of a pneumatic launching system against a steel plate. The effects of impacts with the velocity values in the range of 30–120 m/s were observed, and conclusions concerning the resistance to cracking were formulated.

A considerable increase of the strain rate values is offered by the Split Hopkinson Pressure Bar (SHPB) technique. This method was used for investigating the mechanical response of the JA-2, M30, and XM39 propellants for the strain rate values of several thousand s^−1^ in [14,15]. The results obtained were compared with the results of static compression tests and compression tests for much lower values of the strain rate. The SHPB method was also used for determining the mechanical response of solid rocket propellants, especially of the HTPB type [16,17,18].

From the analysis of the above works, it follows that their authors were interested mainly in investigating the mechanical response of the propellants, without finding any correlations between the results of mechanical tests and the results of ballistic properties testing of the gun propellants. In this work, an attempt is made to find such correlations for single-base, double-base, and LOVA-type propellants.

The mechanical properties of the propellants were tested by using both quasi-static compression with the strain rate value equal to 0.001 s^−1^ and dynamic compression by the use of the SPHB with the strain rate values in the range of 1000–6000 s^−1^. As it is shown in the paper, the load rate values characteristic for the closed vessel tests are intermediate between the rates characteristic for the quasi-static tests and for the SPHB tests. The main objective of the investigation was the verification of the hypothesis that observed differences between the theoretical shape function of propellant grains and the shape function determined on the basis of closed vessel tests results are a consequence of the changed geometry and cracking of propellant grains. So, to verify this hypothesis, the results of mechanical tests were used for analyzing deviations of the experimentally determined shape functions from the theoretical shape functions.

## 2. Materials and Experimental Procedure

### 2.1. Propellant Materials

Three types of propellants were tested: a single-base 12/7 propellant, a double-base JA-2 propellant, and a composite propellant named SC. The first two propellants are commercially available, while the third is laboratory-made.

The 12/7 propellant is used for the elaboration of artillery ammunition of caliber 122 and 152 mm. Its chemical composition is given in Table 1.

The JA-2 propellant, applied for elaboration of a 120 mm tank ammunition, is produced in the surface-coated double-base technology. Its chemical composition is given in Table 2.

The composite propellant SC is designated for the elaboration of low vulnerability ammunition (LOVA). Low vulnerability is connected with the higher ignition temperature and relatively low burning rate at low values of pressure. The main components of the SC propellant are given in Table 3.

The geometrical characteristics of the tested propellants are given in Table 4.

### 2.2. Strength Compression Experiments

#### 2.2.1. Specimen Preparation

Cylindrical specimens of the 12/7, JA-2, and SC propellants were cut from propellant grains using a Proxxon scroll saw. To ensure a mutual parallelism of the specimen ends and their perpendicularity to the specimen axis, a propellant grain was clamped in a miniature vice, which was placed on the scroll saw table and moved only along an adjustable fence during cutting. Additionally, the specimen end surfaces were polished after the cutting process using 500 grit sandpaper. For the quasi-static strength testing, the specimens were trimmed in length to obtain a length-to-diameter ratio (*L*/*D*) of about 1. In turn, the *L*/*D* specimen ratios for high strain rate testing were 0.3, 0.5, and 1, respectively. Many researchers have pointed out that the stress state equilibrium in soft material specimens is hard to achieve for thick specimens deformed with a high strain rate, especially for the first stage of elasto-plastic deformation [21,22]. However, reducing the specimen thickness (decreasing the *L*/*D* ratio) to obtain the stress balance in contact specimens ends may cause a test result error to arise from friction effects [22,23]. Therefore, specimens with different *L*/*D* ratios for the SHPB experiments were prepared to assess the stress state equilibrium of the tested propellant specimens and to select the most appropriate *L*/*D* ratio.

The specimen diameters corresponded to the diameter of the grains of tested propellants and were measured by a digital micrometer with a blade-shaped anvil and non-rotating spindle. A minimum of three measurements of the grain diameter were made to determine the average cross-section area of a given specimen (*A_s_*).

#### 2.2.2. Quasi-Static Compression Testing

In the quasi-static compression tests, an MTS Criterion C45 electromechanical strength machine (Eden Prairie, MN, USA) was used to deform the propellant specimens to a strain of 0.5. The tests were performed at room temperature and under constant velocity of a crosshead, which corresponded to an average strain rate of the order of 0.001 s^−1^. To minimize the friction between the contact surfaces of the compression grips and specimens, and to reduce specimen barreling, molybdenum disulfide (MoS_2_) petrolatum was applied. For each propellant, at least three specimens were tested.

#### 2.2.3. High Strain Rates Compression Testing

**Figure 1:**
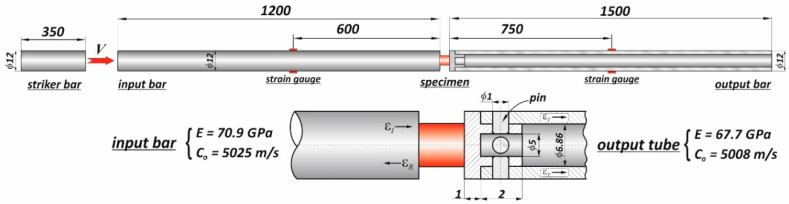
Schematic illustration of the hollow Split Hopkinson Pressure Bar (SHPB) setup.

Gun propellants, from the mechanical point of view, belong to the group of materials called soft materials or low mechanical impedance materials. If a specimen is made from a soft material, application of the SHPB technique needs to solve many technical and methodological problems. These issues were considered in works [23,24,25], among others, where different approaches to studies of soft materials by the SHPB were presented. In the present work, a modified SHPB setup equipped with a hollow output bar with an end cap instead of a traditional solid output bar was used, as shown in Figure 1.

The total length of the SHPB device, which consists of a gas gun system with a striker bar and two long bars, was about 4.5 m. The 350 mm long striker bar and 1200 mm long input bar with a common diameter of 12 mm were made of Al 7075-T6 (Young’s modulus *E* = 70.9 GPa; elastic wave velocity *C*_0_ = 5025 m/s), whereas the 12 mm hollow output bar with a length of 1500 mm and 6.86 mm inner diameter was made of an Al 6063-T53 tube (*E* = 67.7 GPa, *C*_0_ = 5008 m/s). To support the specimen between bar ends correctly, the end cap was pressed into the hollow input tube. Moreover, four small pins with 1 mm diameter were used to keep the end cap in position (Figure 1). The end cap and the pins were made from the same aluminum alloy as the hollow input tube. Applying a hollow bar as the input bar is required due to the problem of a too-small amplitude of the transmitted signal (wave), which is a consequence of a large mismatch of mechanical impedance between the specimen and the metallic bars. The specimen thickness also influences the attenuation of the transmitted signal because the stress wave propagating in soft material has relatively low velocity.

The wave signals in the input and output bars were captured using a pair of strain gauges attached symmetrically to the opposite surfaces of the bars and at their mid-points. Typical electrical strain gauges of 1.6 mm length were used. The amplified signals of the strain gauges were recorded using a data acquisition system with a frequency band of 1 MHz.

To minimize the wave dispersion (Pochhammer–Chree high-frequency oscillations) and to facilitate stress equilibrium, a pulse shaping technique was used. The pulse shaper size has to be chosen for the given striker impact velocity and mechanical response of the tested materials. It was found that for the given SHPB test conditions, the copper pulse shaper with a diameter of 3 mm and thicknesses in the range from 0.2 to 0.3 mm (depending on impact striker velocity *V*) guarantees the damping of high-frequency oscillations and improves the dynamic stress state equilibrium in the specimens.

The impact striker bar velocities applied during the SHPB experiments were in the range of 12 to 23 m/s, which ensure strain rates of 1000–6000 s^−1^ with the specified size of employed specimens. Similarly to quasi-static testing, grease on the basis of molybdenum disulfide (MoS_2_) was applied to the interfaces between the specimen and the bars to minimize the interfacial friction. Moreover, the deformation and/or failure of the tested propellant specimens were recorded with the use of a high-speed camera (Phantom v1612) with a resolution of 384 × 256 and a frame rate of 65,000 frames per second. The high-speed camera system was also applied to acquire a sequence of photographs for determining the relative motion of the specimen‒bar ends interfaces, i.e., the specimen strain, instead of measuring the incident (ε*_I_*) and reflected (*ε_R_*) waves profiles to calculate the nominal strain according to Equation (7) presented in [25]. Such approach resulted from the very large discrepancy found between the nominal strain value determined on the basis of *ε_I_* and *ε_R_* wave profiles and the nominal strain estimated from measuring specimen dimensions after the SHPB experiment. The stress profile at the output bar/specimen interface was determined in the classical way, i.e., in accordance with the SHPB theory [26] based on the transmitted (*ε_T_*) wave signal recorded by the output bar strain gauges.

### 2.3. Closed Vessel Tests and Method of Closed Vessel Test Data Reduction

The closed vessel tests (CVT) were performed in a 200 cm^3^ capacity vessel for two values of the loading density: ∆ = 100 and 200 kg/m^3^. For each value of the loading density, the test was repeated twice. The propellant combustion was initiated by a black powder igniter. The mass of the igniter was chosen so that 3 MPa ignition pressure was attained. The pressure inside the vessel was measured by a 5QP 6000M piezoelectric sensor manufactured by HPI-GmbH. Pressure courses were sampled with a time step equal to 25 μs.

The process of burning of propellants is usually characterized by the vivacity function. However, the vivacity is influenced by many factors, including the energetic characteristics, the size and the shape of propellant grains, and the burning rate. Much more suitable for an analysis of the relations between the mechanical properties of propellants and their burning characteristics is the experimental shape function *ϕ_ex_* introduced in [27]. This function can be compared with the theoretical shape function, i.e., the relation between the relative burning surface *ϕ* and the relative volume *z* of burned propellant. The theoretical shape function is fully determined by the geometry of the propellant grains. Differences between theoretical and experimental shape functions provide information about changes of the geometry of grains caused by deformation and cracking under high pressure.

The method of calculating the theoretical shape function is well described in the literature [1]. The experimental shape function is determined based on the pressure records *p*(*t*) determined in closed vessel tests. The records are smoothed by the Loess algorithm [28] and the pressure time derivative d*p*/d*t* is calculated. Based on the Noble–Abel equation of state, the value of the relative volume of burned propellant and its pressure derivative are calculated [1,2,3]:

(3) $$z=b_{1}p_{s}/\left(f+b_{2}p_{s}\right),$$

(4) $$dz/dp=\left(b_{1}f\right)/{\left(f+b_{2}p_{s}\right)}^{2},\;p_{s}=p-p_{i},\;b_{1}=1/\Delta -1/\rho ,\;b_{2}=\eta -1/\rho .$$

The parameter *f* is the propellant force, *p_i_* is the ignition pressure, *ρ* is the propellant density, *η* is the co-volume, and ∆ is the loading density of the propellant charge. The time derivative of the relative volume of the burned propellant is calculated:

(5) dz/dt=(dz/dp)(dp/dt).

The physical burning law [1,27] gives the following relation:

(6) $$dz/dt=G\left(z\right)p_{0}x^{n},\;x=p/p_{0},\;p_{0}=0.1\;\mathrm{MPa}.$$

The function *G*(*z*), introduced in [27], is a characteristic of a given propellant. It is a generalization of the function *Г*(*z*) introduced in [1] for the linear dependence of the burning rate on pressure. It is used for the general power dependence of the burning rate on pressure. From Equation (6), we obtain:

(7) $$\log_{10}\left(dz/dt\right)=\log_{10}\left[G\left(z\right)p_{0}\right]+n\log_{10}x\;.$$

By calculating the d*z*/d*t* values for several values of the loading density and the same value of *z*, we obtain a linear relation between the log_10_(d*z*/d*t*) values and log_10_(*x*) values. The slope determines the value of *n*; thus, the *n* values for *z* = 0.3, 0.4, 0.5, 0.6, and 0.7 are determined, and the average value is calculated. Then, values of the function *G*(*z*) are calculated:

(8) $$G\left(z\right)=\left(dz/dt\right)/\left(p_{0}x^{n}\right).$$

There is the following relation between the function G(z) and the experimental shape function [27]:

(9) $$\phi_{ex}\left(z\right)=\frac{G\left(z\right)}{\theta },\;\theta =\beta p_{0}^{n-1}S_{0}/V_{0}.$$

*β* is the coefficient in the burning rate law, while *S*_0_ and *V*_0_ are the initial values of the grain surface and the grain volume. The coefficient *β* is calculated by the formula [27]:

(10) $$\theta =J_{1}/\left(p_{0}J_{2}\right),\;J_{1}=\int_{z_{1}}^{z_{2}}1/\phi_{ex}\left(z\right)dz,\;J_{2}=\int_{t_{1}}^{t_{2}}x^{n}dt,$$

where *z*_1_ = 0.3 and *z*_2_ = 0.7 were assumed. 

The values of *t*_1_ and *t*_2_ correspond to these values of *z*. Assuming that the integral *J*_1_ in Equation (10) can be approximately estimated by replacing *ϕ_ex_*(*z*) with ϕ (*z*), we can use the calculated *θ* value and determine *ϕ_ex_*(*z*) based on Equation (9). The integral *J*_1_ is calculated again, and a new value of *θ* is determined. We repeat this procedure until a satisfactory convergence of *θ* values is attained and the final experimental shape function is obtained.

## 3. Results and Discussion

### 3.1. Mechanical Testing Analysis

**Figure 2:**
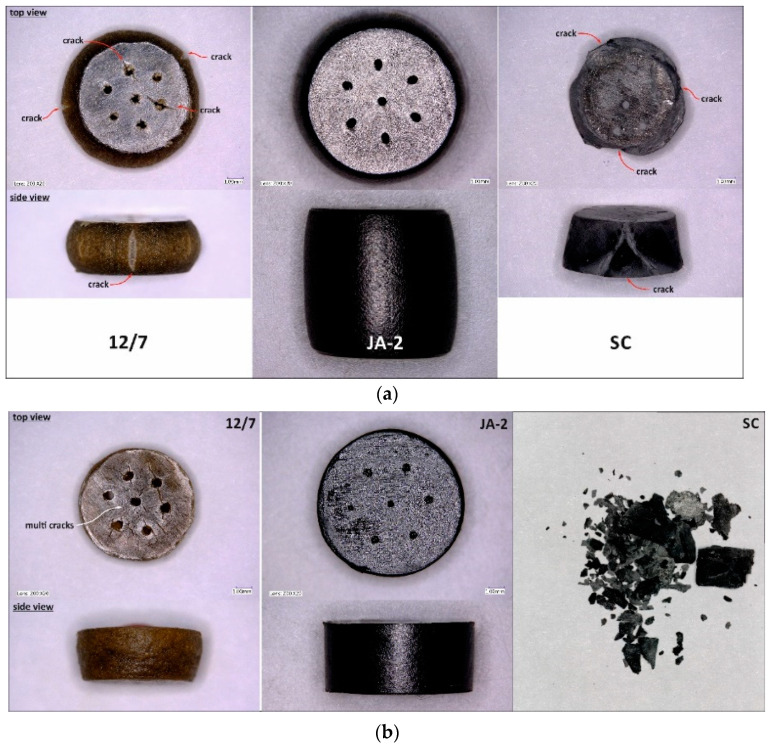
Propellant specimens after quasi-static compression (**a**) and SHPB tests (**b**).

**Figure 3:**
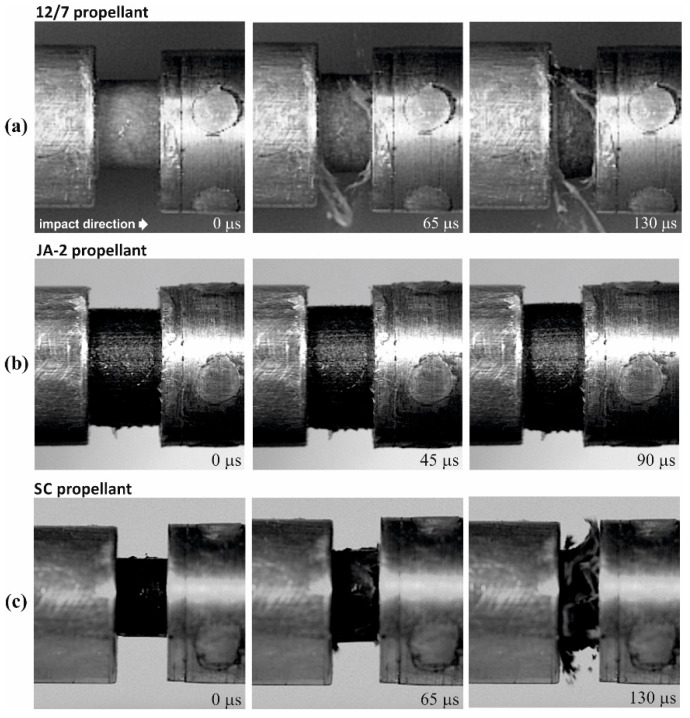
High-speed images of the tested propellant specimens deformed under the SHPB testing condition: (**a**) 12/7 propellant (*V* = 23.0 m/s; $\dot{\varepsilon }=2870\;s^{-1}$), (**b**) JA-2 propellant (*V* = 12.1 m/s; $\dot{\varepsilon }=1920\;s^{-1}$), (**c**) SC propellant (*V* = 12.0 m/s; $\dot{\varepsilon }=2120\;s^{-1}$).

Views of propellant grains after quasi-static compression are shown in Figure 2a. The samples of the JA-2 propellant remained intact, while cracks were observed in the case of the 12/7 and SC propellants. The JA-2 samples subjected to the dynamic load also remained intact (see Figure 2b), whereas multi-cracks were observed in the case of 12/7 propellant. The specimens of the SC propellant were completely crushed, as shown in Figure 2b and Figure 3, in which three selected frames of the high-speed camera record are presented.

**Figure 4:**
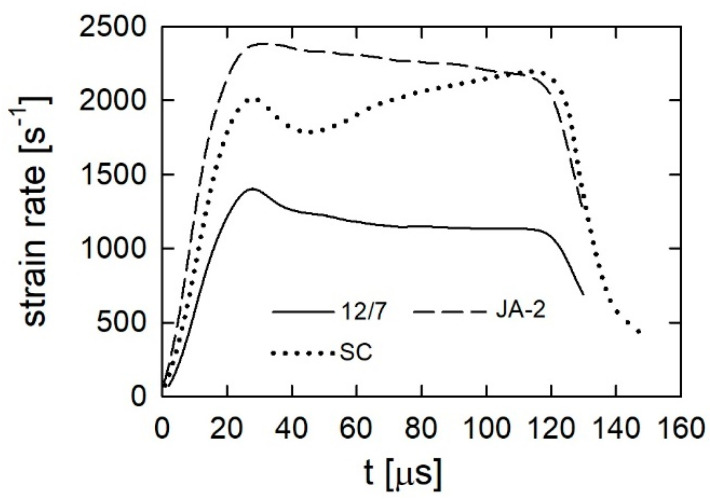
Strain rate histories in the SHPB experiments.

**Figure 5:**
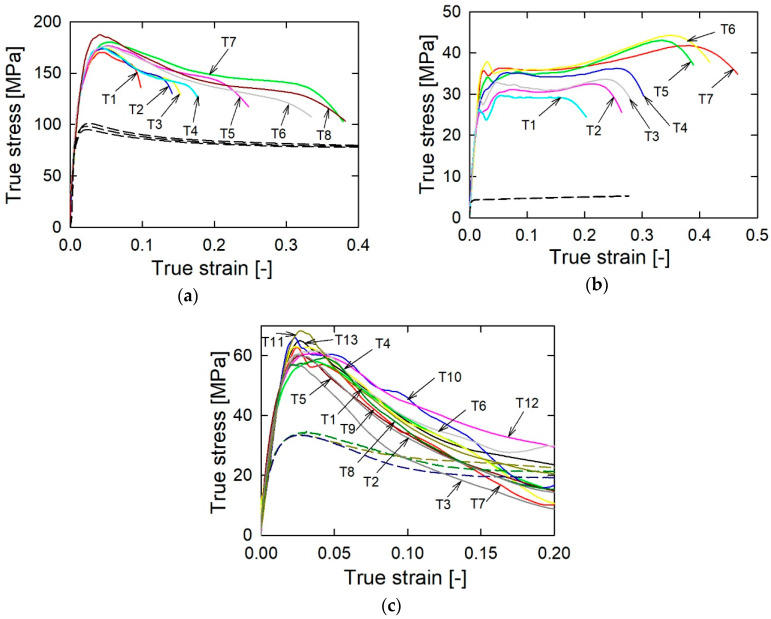
True stress‒strain curves for the 12/7 (**a**), JA-2 (**b**), and SC (**c**) propellants: solid lines–SHPB tests, dashed lines–quasi-static tests.

In turn, Figure 4 presents examples of strain rate curves for each propellant obtained from the SHPB experiments. The strain rate value corresponding to the first maximum is close to the value at which the yield of propellant materials takes place. The second maximum in the SC propellant case corresponds to total crushing of the propellant grains. Therefore, we decided to characterize the tests by values corresponding to the first maximum of the strain rate. These are given in Table 5. In the quasi-static test, the strain rate value was of the order of 0.001 s^−1^. Figure 5a–c present the true stress–strain plots obtained from the quasi-static and the SHPB tests.

The single-base 12/7 propellant shows the highest values of the yield stress in both the quasi-static and dynamic experiments. In turn, the JA-2 propellant is the softest of the tested propellants, and it reveals the highest strain rate sensitivity. The SC propellant has intermediate values of the yield strength. Both the 12/7 and SC propellants show a modest strain rate sensitivity. Moreover, the JA-2 propellant is characterized by the almost perfectly plastic behavior after the yield (low strain-hardening effect). In turn, the 12/7 and SC propellants reveal mechanical weakness i.e., decreasing flow stress with increasing strain, similar to the strain-softening behavior observed in some metal alloys [29]. However, in the case of the present work, the mechanical weakness of the 12/7 and SC propellant specimens can be explained by the cracking. It should be noted here that in the case of the 12/7 propellant, the specimens remain intact despite the cracking. This is reflected in the regular shape of the descending parts of the stress‒strain plots. In turn, the descending parts of the stress‒strain curves for the SC propellant show a great scatter. This reflects the random character of the damage process of the grain specimens.

**Figure 6:**
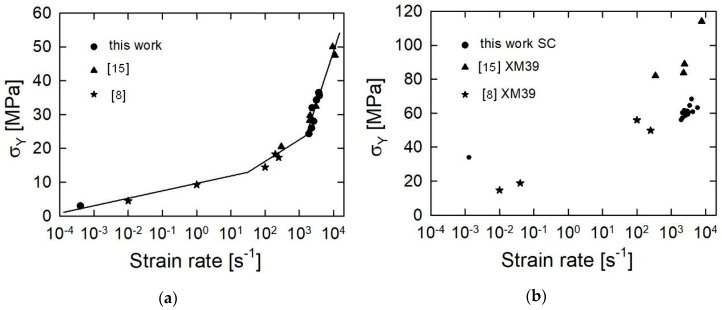
Strain rate dependence of the flow stress for the JA-2 (**a**) and for XM39 and the SC (**b**) propellants: solid lines–approximation (11).

The results obtained for the JA-2 propellant can be compared with the results obtained in [15], where the SHPB test results for this propellant are reported. Figure 6a presents a strain rate dependence of the visco-plastic flow stress for the JA-2 propellant. The values of the flow stress determined in [15] and in this work are shown. Moreover, the results of the drop weight tests from [8] are also shown. The results of the present work agree well with the results of the SHPB tests obtained in [15].

As was shown in Figure 6a, the strain rate dependence of the flow stress can be approximated by the following relation:

(11) $$\sigma_{Y}=\{\begin{array}{c} 9.65+0.94\log\left(\dot{\varepsilon }/{\dot{\varepsilon }}_{0}\right)\;\dot{\varepsilon }\in \left[0.0001,31.4\right]\;\\ 9.65+2.17\log\left(\dot{\varepsilon }/{\dot{\varepsilon }}_{0}\right)\;\dot{\varepsilon }\in \left[31.4,1580\right]\\ -74.3+19.3\log\left(\dot{\varepsilon }/{\dot{\varepsilon }}_{0}\right)\;\dot{\varepsilon }\in \left[1580,15000\right] \end{array}{\dot{\varepsilon }}_{0}=1s^{-1},\;[\dot{\varepsilon }]=s^{-1},\;[\sigma_{Y}]=\mathrm{MPa}.$$

In turn, data for the LOVA-type XM39 propellant published in [8,15] are shown in Figure 6b together with data determined in the present work for the SC propellant. Although both propellants are of the same type, their mechanical properties differ considerably. The SC propellant has much greater strength for low strain rates and is much less strain rate sensitive. However, the two propellants show a brittle behavior, and this is manifested by the negative slopes of the visco-plastic parts of the *σ*(ε) curves.

### 3.2. Analysis of Closed Vessel Tests Results

**Figure 7:**
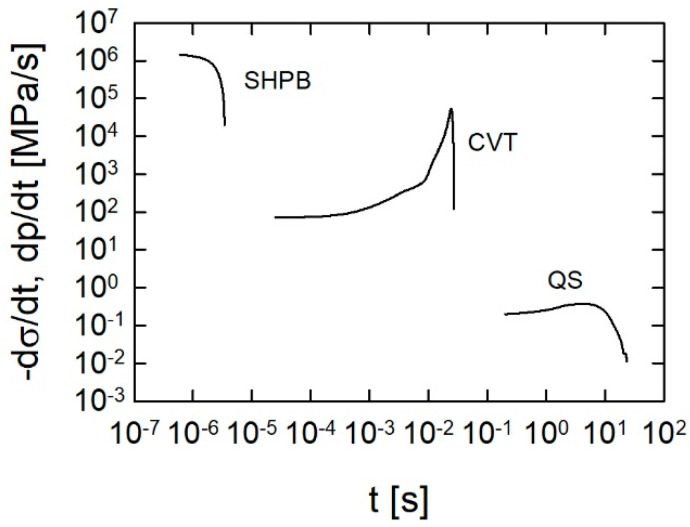
Load rates levels for the quasi-static (QS), closed vessel (CVT), and SHPB tests.

A comparison of the scale of the load rates in the quasi-static, closed vessel, and SHPB tests is given in Figure 7 for the JA-2 propellant. The SHPB test with the lowest strain rate was chosen. It follows from Figure 7 that the load rates for the closed vessel tests are intermediate between the characteristic rates of stress for the SHPB and the quasi-static experiments. This means that based on the results of the dynamic and quasi-static strength tests, we can draw inferences concerning the mechanical behavior of the propellants in closed vessel tests.

**Figure 8:**
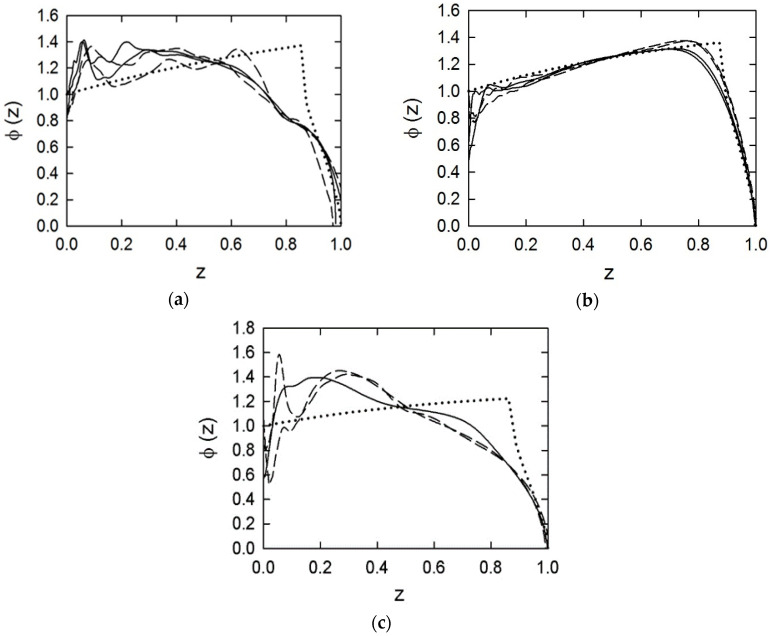
Shape function for the 12/7 (**a**), JA-2 (**b**), and SC (**c**) propellants: solid lines ∆ = 200 kg/m^3^, dashed lines ∆ = 100 kg/m^3^, dotted line—theoretical shape function.

Table 6 presents the values of the propellant force *f*, the covolume *η*, and the exponent in the burning rate law *n*. Two sets of parameters values are presented: without correction for heat losses and corrected values. Using these values, the experimental shape functions were calculated. The plots of the shape functions are presented in Figure 8a–c.

There is a clear correspondence between the results of the investigations of the mechanical properties of the tested propellants and their shape functions. The JA-2 propellant, showing plastic behavior and remaining intact under the loading, has a shape function that deviated very little from the theoretical shape function. The observed deviations can be attributed to effects of the ignition. Propellant grains are not ignited at the same time. Therefore, some of them end up burning earlier than others. This causes the transition to the degressive phase of burning to begin earlier than in the case when all the grains are ignited at the same time, as is assumed in calculations of the theoretical shape function.

In the case of the 12/7 and SC propellants, the relative burning surface exceeds the nominal relative burning surface by 20–30%. This means that during the burning process, an additional surface is created due to cracking of the propellant grains. The fragments created have shapes that cause the burning surface to decrease with the increasing volume of burned propellant. This means that the burning process has a degressive character. In the geometric law, the degressive phase of burning of seven-perforated propellants begins for *z* = 0.87. In the closed vessel tests, this phase begins at *z* = 0.7–0.8 for the JA-2 propellant, at *z* = 0.4–0.6 for the 12/7 propellant, and at *z* = 0.2–0.3 for the SC propellant.

The physical burning law, Equation (6), assumes that there is no dependence of the burning surface on the pressure value. From this assumption, it follows that no differences between the shape functions determined for different values of the loading density occur. In the case of the JA-2 propellant, there are only slight differences. In the case of the 12/7 and the SC propellants, these differences are pronounced.

**Figure 9:**
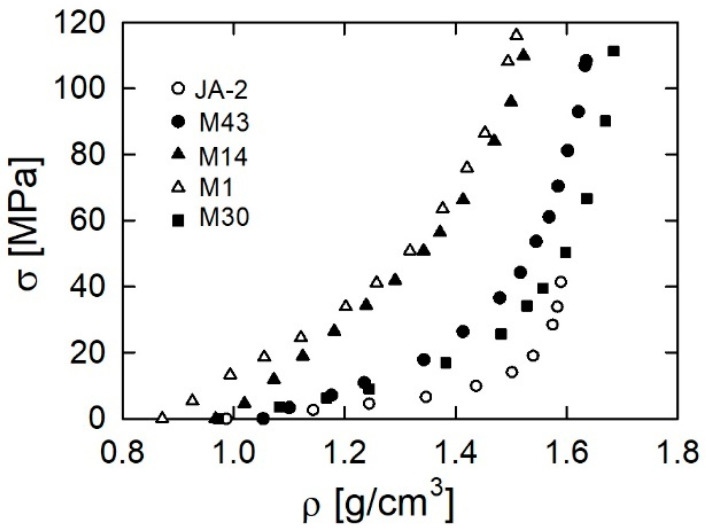
Dependence of the axial stress on the density of the propellant bed; experimental data from Figures 2 and 3 in [30].

Is it justified to use the results of uniaxial compression tests to explain the behavior of propellants in closed vessel tests? Theoretically, the mechanical loading of propellant grains in the closed vessel tests is isostatic, because the grains are loaded by the pressure of the surrounding hot gases. However, there are some deviations from loading of this character. When the propellant bed is ignited, the pressure acts on a bed of unignited grains, so the load is not isostatic. The behavior of propellant beds under quasi-static uniaxial loading was investigated in [30]. Beds of the single-base propellants M1 and M14, double-base propellant JA-2, triple-base propellant M30, and composite propellant M43 were loaded. Figure 9 presents plots of the dependence between the density of a propellant bed and the stress. The behavior of the beds of propellants is just as can be expected based on the results of uniaxial loading of propellant grains presented in Section 3.1. The single-base propellants show the highest resistance to the load, while the JA-2 propellant shows very low resistance. The composite propellant M43 shows an intermediate resistance.

When the propellant grains are fully ignited, the value of the pressure inside the perforation may exceed the pressure acting on the outer surface. This is caused by a delay in evacuation of the gases produced from the perforation. The higher value of the pressure inside the perforation accelerates the burning rate. As a result, the propellant burns faster, as in the case when the burning rates on the outer and inner surfaces are the same. Apparently, this is equivalent to an increase of the burning surface. This effect was pointed out in [1] as a reason for deviations from the geometric burning law. However, this effect is not observed in the case of the JA-2 propellant. We can attribute this to the mechanical properties of this propellant. The higher value of the pressure inside the perforation may cause an increase in the diameter of perforation, which facilitates the evacuation of gases. So, the deformation of grains compensates for the effect of accelerated burning of the inner surfaces. However, despite the deformation, the grains remain intact, and no additional surface is created. For the 12/7 and SC propellants, the brittle behavior was observed. This means that the deformation may be accompanied by cracking.

Two other factors should be taken into account in explaining the results of the closed vessel tests. The magnitude of the stresses inside the propellants grains depends on the deviations from the symmetry of the grains: the larger the deviations, the higher the stresses. The JA-2 propellant grains have a perfect shape, while the shapes of the laboratory-made SC propellant grains show relatively large deviations from the ideal shape. This applies to a lesser extent to the 12/7 propellant. Higher inner stresses in materials prone to brittle behavior may be the reason for the observed increase in the burning surface.

**Figure 10:**
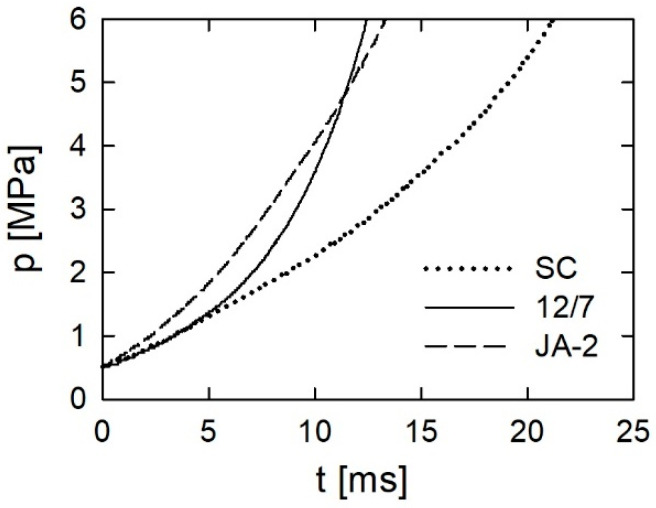
Initial parts of pressure records for ∆ = 100 kg/m^3^.

The ignition temperature is the second factor that may have an influence on the behavior of propellants in closed vessel tests. It is the lowest for the JA-2 propellant, higher for the 12/7 propellant, and the highest for the SC propellant. The higher the ignition temperature, the longer the ignition time. This can be illustrated by the initial fragments of pressure records shown in Figure 10. The moment when the value of 0.5 MPa is attained is chosen as a starting time. The ignition time of the JA-2 propellant is the shortest, while the ignition time of the SC propellant is the longest. If a propellant is difficult to ignite, a situation is possible when only a part of the perforation of the grains is ignited. In this case, the loading scheme of propellant grains deviates considerably from the isostatic one and may cause the cracking of grains. This factor does not act in favor of the SC propellant.

The prolonged ignition process may cause heating of the outer parts of grains. This causes an accelerated burning of these parts. This effect was considered in [31,32] as a reason for deviations from the geometric burning law. The initial peaks of the *ϕ* values in Figure 8a–c may be attributed to this effect. However, this effect is important for propellant grains with low web values. The grains of the tested propellants had relatively high web values. In the course of the burning process, the initially heated layers are burned. So, after a transient faster burning, the burning process should obey the geometric law of burning. Instead of this, degressive burning was observed for the 12/7 and SC propellants. This can be explained only by the crushing of propellant grains.

## 4. Conclusions

Juxtaposition of the results of mechanical tests and closed vessel tests confirmed the correctness of the hypothesis that the observed deviations of the burning process from the geometrical burning model can be attributed mainly to the cracking of propellant grains. This shows that the mechanical properties of the propellants are important not only from the point of view of logistical issues but also for the proper functioning of propellants during the shot. It may seem counterintuitive, but relatively soft propellants, showing visco-plastic behavior and high strain rate sensitivity, burn in a way that is close to the geometric law. Relatively rigid single-base propellants may undergo cracking, which makes the process of burning deviate from the geometric law. The crushing of grains may be a serious problem for composite propellants. This means that the rheological properties of these propellants may determine their usability.

## Figures and Tables

**Table 1:** Percentage composition of the 12/7 propellant.

NC ^1^	DFA ^2^	Volatiles
95.2	1.5	3.3

^1^ NC–nitrocellulose, ^2^ DFA–diphenylamine.

**Table 2:** Percentage composition of the JA-2 propellant [19].

NC ^1^	NG ^2^	DEGDN ^3^	Akardite II	Graphite
59.5	14.9	24.8	0.7	0.1

^1^ NC–nitrocellulose, ^2^ NG–nitroglycerin ^3^ DEGDN–diethylene glycol dinitrate.

**Table 3:** Percentage composition of the SC propellant.

RDX ^1^	NC ^2^	CAB ^3^	TAC ^4^	Other
75	10	6	7	2

^1^ RDX–hexogen, ^2^ NC–nitrocellulose, ^3^ CAB–cellulose acetate butyrate ^4^ TAC–triallyl cyanurate.

**Table 4:** Geometrical characteristics of tested propellant grains.

Parameter	12/7	JA-2 [20]	SC [20]
Grain shape	cylindrical	cylindrical	cylindrical
Perforation number	7	7	7
Length *L* [mm]	14.55	15.5	8.7
Diameter *D* [mm]	6.6	8.8	6.3
Perforation diameter *d* [mm]	0.6	0.55	0.58
Web thickness [mm]	1.2	1.78	1.14

**Table 5:** Strain rate values in SHPB tests [s^−1^].

Test	T1	T2	T3	T4	T5	T6	T7	T8	T9	T10	T11	T12	T13
**12/7**	1090	1420	1720	1730	1980	2540	2730	2870	-	-	-	-	-
**JA-2**	1920	2270	2380	2660	3170	3710	3900	-	-	-	-	-	-
**SC**	1950	2110	2120	2410	2610	2780	2920	3040	3190	3450	3920	4250	5820

**Table 6:** Values of the propellant force, the covolume, and the exponent in the burning rate law determined without correction for heat losses (**E**) and corrected (**Q**).

Parameter	12/7	JA-2 [20]	SC [20]
Force (E) [kJ/kg]	1096	963	968
Force (Q) [kJ/kg]	1260	1167	1113
Covolume (E) [dm^3^/kg]	0.967	1.465	1.608
Covolume (Q) [dm^3^/kg]	0.516	1.011	1.233
Exponent (E)	0.779	0.841	0.872
Exponent (Q)	0.898	1.014	0.980
